# Editorial: Advances in Mathematical and Computational Oncology

**DOI:** 10.3389/fphys.2022.889198

**Published:** 2022-04-07

**Authors:** George Bebis, Doron Levy, Russell Rockne, Ernesto Augusto Bueno Da Fonseca Lima, Panayiotis V. Benos

**Affiliations:** ^1^ Department of Computer Science and Engineering, University of Nevada, Reno, Reno, NV, United States; ^2^ Department of Mathematics, University of Maryland, College Park, MD, United States; ^3^ Department of Computational and Quantitative Medicine, Beckman Research Institute, City of Hope, Pasadena, CA, United States; ^4^ Center for Computational Oncology, Oden Institute for Computational Engineering and Sciences, University of Texas at Austin, Austin, TX, United States; ^5^ Department of Computational and Systems Biology, University of Pittsburgh, Pittsburgh, PA, United States

**Keywords:** mathematical oncology, computational oncology, machine learning, mathematical modeling, computational modeling

Cancer is not a single disease, it is a complex and heterogeneous disease which leads to the second cause of death worldwide. Although all cancers manifest themselves as an uncontrolled growth of abnormal cells, they are actually distinct neoplastic diseases that possess different genetic and epigenetic alterations, underlying molecular mechanisms, histopathologies and clinical outcomes. Understanding the origins and growth of cancer requires understanding the role of genetics in encoding proteins that form phenotypes and molecular alterations at multiple levels (e.g., gene, cell, and tissue).

Advanced mathematical and computational models could play a significant role in examining the most effective patient-specific therapies. Tumors, for example, undergo dynamic spatio-temporal changes, both during their progression and in response to therapies. Multiscale advanced mathematical and computational models could provide the tools to make therapeutic strategies adaptable enough and to address the emerging targets. Similarly, understanding the interrelationship amongst complex biological processes requires analyzing very large databases of cellular pathways. High-performance computing, big data analytics solutions, data-intensive computing, and medical image analysis techniques could be critical in addressing these challenges. Therefore, there is pressing need to design and develop mathematical and computational strategies to harness cancer data in an accurate and efficient fashion.

This special issue includes contributions to the state of the art and practice in mathematical and computational oncology addressing some of the challenges and difficulties in this field, as well as prototypes, systems, tools, and techniques.

Identifying potential biomarkers with prognostic value for various cancers has been a challenging research problem. Xu et al. (Screening and Identification of Potential Prognostic Biomarkers in Adrenocortical Carcinoma) investigate this problem in silico for the case of adrenocortical carcinoma (ACC) by integrating protein interaction networks with gene expression profiles. By looking for the most significantly differentially expressed genes in three microarray datasets from the Gene Expression Omnibus (GEO) database, they identified 150 genes that overlapped in the three datasets. These 150 significant genes were further analyzed using DAVID, KEGG and other methods resulting in 24 hub-genes, which were then used for downstream analysis and validation. Using these 24 hub-genes for Disease Free Survival (DFS) and Overall Survival (OS) in a cohort of 76 ACC cases from the TCGA revealed that 5 of these hub-genes were significantly correlated with either DFS or OS. By performing univariate and multivariate Cox regression, as well as Kaplan-Meier survival analysis, the authors demonstrated the potential prognostic value of the mRNA overexpression of these 5 genes in ACC.

In an effort to better understand why rectal cancer patients, even with the same tumor stage, have different response to radiotherapy, Pham et al. (Image-Based Network Analysis of DNp73 Expression by Immunohistochemistry in Rectal Cancer Patients) investigate the predictive value of DNp73 in patients with rectal adenocarcinoma using image-based network analysis. Using Fuzzy Weighted Recurrence Networks (FWRN), they analyzed the immunohistochemistry images of DNp73 expression from a small cohort of rectal cancer patients who underwent radiotherapy before surgery. Their analysis showed that the primary tumors-to-biopsy ratios of two FWRN parameters, namely the clustering coefficient and the characteristic path length, can correlate DNp73 expression with increased survival time.

McKenna et al. (Leveraging Mathematical Modeling to Quantify Pharmacokinetic and Pharmacodynamic Pathways: Equivalent Dose Metric) provide a framework for leveraging mathematical modeling to quantify pharmacokinetic and pharmacodynamic (PK/PD) pathways. Standard treatment response assays compare cell survival at a single timepoint to applied drug concentration overlooking drug PK/PD properties in developing treatment response assays. Addressing this oversight, McKenna et al. utilize mathematical modeling to decouple and quantify PK/PD pathways. They propose a notion of an “equivalent dose metric”, a metric that is derived from a mechanistic PK/PD model and provides a biophysically-based measure of drug effect. An equivalent dose is defined as the functional concentration of drug that is bound to the nucleus following therapy. This metric can be used to quantify drivers of treatment response and potentially guide dosing of combination therapies. Examples are provided through studying the response of cells to time-varying doxorubicin treatments, modulating doxorubicin pharmacology with small molecules that inhibit doxorubicin efflux from cells and DNA repair pathways. This approach can be leveraged to quantify the effects of various pharmaceutical and biologic perturbations on treatment response.

Evaluating the dynamics interactions of multiple angiogenic factors involved in tumor angiogenesis was attempted by Li and Finley (Exploring the Extracellular Regulation of the Tumor Angiogenic Interaction Network Using a Systems Biology Model). In this context, they propose a new computational framework to understand the extracellular distribution of angiogenic factors in tumor tissue and generate new insights into the regulation of the angiogenic factors’ interaction network. The model describes the distribution of two potent pro-angiogenic factors and two important anti-angiogenic factors in tumor tissue. The model predicts that most of VEGF and FGF2 is bound to the cell surface and in signaling forms, while most of TSP1 and PF4 is in the interstitial space and in non-signaling forms that are trapped by HSPGs or inactive due to proteolysis. Moreover, it predicts that increasing the secretion of PF4 in tumor tissue can lead to two counterintuitive results: an increase in interstitial FGF2 and VEGF levels and greater formation of pro-angiogenic complexes, particularly in the VEGF signaling pathway. The study provides mechanistic insights into these counterintuitive results and highlights the role of heparan sulfate proteoglycans in regulating the interactions between angiogenic factors.

SNARE proteins facilitate membrane fusion and as such they have a multifaceted role in the life of cells and have been associated to multiple diseases, including cancer. Thus, identifying SNARE proteins from sequence composition only has become quite important. Previous methods rely on motif identification and some use position-specific scoring matrices, obtained from alignments, as (image equivalent) inputs to a 2D convolutional neural network (CNN). Le and Huynh (Identifying SNAREs by Incorporating Deep Learning Architecture and Amino Acid Embedding Representation) propose a new method for identifying new SNARE proteins. First, they used protein motif information to extract 26,789 non-redundant “SNARE superfamily” proteins from NCBI; and equal number of non-SNARE proteins. Second, they used the NLP algorithm “fastText” (used by FaceBook) to create amino acid embedding representations. Finally, they used these representations as input to a 1D CNN to predict whether a protein belongs to the SNARE family or not. Given that the peptide “words” do not contain “spaces” like in human languages, they tested n-grams of size *n* = 1,2,3,4,5. Unsurprisingly, they found that *n* = 4 or 5 achieved the best performance. They used *n* = 5 results to compare their method to the other CNN based on position-specific scoring matrices and they found to perform substantially better across metrics (sensitivity, specificity, accuracy, MCC).

Hochman et al. (Metastases Growth Patterns in vivo—A Unique Test Case of a Metastatic Colorectal Cancer Patient) explore colorectal cancer (CRC) lung metastases growth patterns. Available mathematical tumor growth models rely mainly on primary tumor data, and rarely relate to metastases growth. The study is based on a data set of a metastatic CRC patient, for whom 10 lung metastases were measured while untreated by seven serial computed tomography (CT) scans, during almost 3 years. Three mathematical growth models (Exponential, logistic, and Gompertzian) were fitted to the actual measurements. The study explores factors affecting growth pattern including size, location, and primary tumor resection. This study provides evidence that exponential growth of CRC lung metastases is a reliable approximation, and encourages focusing research on short-term effects of surgery on metastases growth rate.

Zhu et al. (Genetic Alterations and Transcriptional Expression of m6A RNA Methylation Regulators Drive a Malignant Phenotype and Have Clinical Prognostic Impact in Hepatocellula) study how genetic alterations and transcriptional expression of m6A RNA methylation regulators derive a malignant phenotype and have clinical prognostic impact in hepatocellular carcinoma (HCC). This study is conducted on data collected from 371 HCC patients from the Cancer Genome Atlas database. Techniques used are survival analysis and gene set enrichment analysis. Machine-learning tools were used on selected regulators to develop a risk signature, m6Ascore. This score is based on four m6A regulators, predicting HCC prognosis well at three or five years. Zhu et al. further show that mutations and copy number variations of m6A regulators, conferring worse survival, are strongly associated with TP53 mutations in HCC.

The ARF/MDM2/p53 is one of the regulatory networks that is heavily solicited in cancer therapy, thus there are important challenges for controlling the output of this network (high p53, low mdm2 with desirable consequences on cell fate). In Suarez et al. (Pinning Control for the p53-Mdm2 Network Dynamics Regulated by p14ARF), a mathematical model of p53-Mdm2 dynamics is used to explore how “pinning” of p14ARF can enable control of gene regulatory network dynamics under biological contexts. The pinning is introduced using a control systems approach where the control dynamics is solved in conjunction with the systems dynamics. In this context, the control system takes the place of either an external perturbation such as DNA damage or an internal reset due to transcription (gene expression). They tested their methodology to confirm 1) the behaviors induced by p53 as DNA damage response to gamma radiation and apoptosis, and 2) the behavior consequence on mdm2 levels and the feedback regulation of p53 levels by mdm2 mediated degradation. Using this approach, the authors propose to computationally model and stir the dynamics of a gene regulatory network such as the p14ARF/MDM2/p53 network to a desired state particularly to reproduce either the coordinated fluctuation behavior of p53 transcription that is usually generated by irradiation and/or to recover the tumor suppressor effect of p53 (cell cycle arrest and apoptosis).

Clear cell renal cell carcinoma (ccRCC) is the most common kidney cancer. Its 5-years survival prognosis increases by 5-fold (from 10% to 50–69%) if it is diagnosed early, when the cancer is small. Therefore, identifying biomarkers of ccRCC is very important. In their study, Yang et al. (Identification of KIF18B as a Hub Candidate Gene in the Metastasis of Clear Cell Renal Cell Carcinoma by Weighted Gene Co-expression Network Analysis) perform a bioinformatic analysis on publicly available gene expression dataset to identify such biomarkers. Specifically, they used a GEO dataset with 265 samples, and after pre-processing and quality control (during which they excluded samples with no meta-data and outliers), they used the popular weighted gene co-expression network analysis (WGCNA) package to identify cancer stage-related gene modules and corresponding “hub” genes. Overall, they identified 10 such genes with high maximal clique centrality (MCC), and KIF18B was at the top of this list. They then validated this finding on the TCGA database where they did not only found KIF18B to be differentially expressed ccRCC patients and controls, but they also found that its high expression was significantly associated with worse survival.

Yu et al. (Predicting Relapse in Patients with Triple Negative Breast Cancer (TNBC) Using a Deep-Learning Approach) performed a preliminary study on predicting the risk of relapse for patients with Triple Negative Breast Cancer (TNBC) using machine learning (ML). By examining the spatial distribution of CD8^+^ T cells and cancer cells in immunofluorescence (IF) images, they derived a prognostic score for predicting early relapse. Using a small dataset, the authors demonstrated that the relative infiltration of CD8 cells into cancer cell islands is associated with good prognosis. The approach could possibly be generalized to other types of cancers.

In every computational model, parameter estimation is an important component. This is also true in systems biology, where biological processes are represented by a set of ordinary differential equations (ODEs). Bianconi et al. (A New Bayesian Methodology for Nonlinear Model Calibration in Computational Systems Biology) present a new Bayesian method for parameter estimation, named Conditional Robust Calibration (CRC). The authors consider the parameter vector as a random variable in the parameter space. The way that CRC works is that it first simulates a fixed number of samples, given a parameter vector. Then it calculates the posterior of the parameters given the data and compares this to the posterior of the observed data. In the next iteration, the parameter vector changes based on the distance (error) of the two. They benchmarked CRC against three other state-of-the-art algorithms (ABC-SMC, profile likelihood, DRAM) on two different systems (Lotka-Voltera model, EpoR system, multiple myeloma model). In the Lotka-Voltera model, all algorithms performed well, with CRC being more accurate although it required one more iteration. In the EpoR system, the CRC found a different solution thatn the other two algorithms, but the authors report their solution was more reliable because of many missing values in this dataset. Finally, the multiple myeloma system is a high-dimensional ODE model. In that system the authors showed that their method outperformed the others.

Storey et al. (Modeling Oncolytic Viral Therapy, Immune Checkpoint Inhibition, and the Complex Dynamics of Innate and Adaptive Immunity in Glioblastoma Treatment) propose an ordinary differential equation model of treatment for a lethal brain tumor, glioblastoma, using an oncolytic Herpes Simplex Virus. Thea authors use a mechanistic approach to model the interactions between distinct populations of immune cells, incorporating both innate and adaptive immune responses to oncolytic viral therapy (OVT), and including a mechanism of adaptive immune suppression via the PD-1/PD-L1 checkpoint pathway. They focus on the tradeoff between viral clearance by innate immune cells and the innate immune cell-mediated recruitment of antiviral and antitumor adaptive immune cells. The model suggests that when a tumor is treated with OVT alone, the innate immune cells’ ability to clear the virus quickly after administration has a much larger impact on the treatment outcome than the adaptive immune cells’ antitumor activity. Even in a highly antigenic tumor with a strong innate immune response, the faster recruitment of antitumor adaptive immune cells is not sufficient to offset the rapid viral clearance. This motivates the subsequent incorporation of an immunotherapy that inhibits the PD-1/PD-L1 checkpoint pathway by blocking PD-1, which is combined with OVT within the model. The combination therapy is most effective for a highly antigenic tumor or for intermediate levels of innate immune localization. Extreme levels of innate immune cell activity either clear the virus too quickly or fail to activate a sufficiently strong adaptive response, yielding ineffective combination therapy of GBM. This work shows that the innate and adaptive immune interactions significantly influence treatment response and that combining OVT with an immune checkpoint inhibitor expands the range of immune conditions that allow for tumor size reduction or clearance.

In the work by Conforte et al. (Modeling Basins of Attraction for Breast Cancer Using Hopfield Networks), bulk RNA-Seq data from 70 paired breast cancer and control samples were analyzed with Hopfield network modeling. Hopfield networks are a form of recurrent artificial neural network which does not require kinetic parameter rates or knowledge of underlying protein-protein interactions. The authors leverage these properties to analyze the high-dimensional RNA-Seq data to find a correlation with the distance between the cancer and control attractors with overall survival. They then use the Hopfield network to identify potential therapeutic gene targets and validate their approach with single-cell sequencing data collected from HER2+ breast cancer patients. This work showcases the power of a theory-driven approach to analysis of complex gene sequencing data and predicts novel therapeutic interventions.

In their paper, Chen et al. (Bioinformatics Analysis of Prognostic miRNA Signature and Potential Critical Genes in Colon Cancer) report the results of bioinformatic analysis they performed in two omics colon cancer datasets. Specifically, they analyzed data form TCGA and another publicly available GEO dataset and identified an 8-miRNA signature predictive of colon cancer prognosis and 14 (mRNA) genes that seem to play a critical role in carcinogenesis. Differential gene/miRNA expression analysis, initially, identified 472 miRNAs and 563 mRNAs that vary significantly between cancer and controls. Further Cox regression analysis found 12 of those miRNAs and 8 of them were used to build the predictive signature (AUROC = 0.729). These 8 miRNAs have a total of 112 target genes, which are also differentially expressed. Downstream pathway analysis for these 112 genes was performed using pathway enrichment and WGCNA. Finally, protein-protein interaction analysis identified 14 of these genes as critically important for colon cancer.

A central challenge of mathematical and computational oncology is the prediction of response to therapy, which often hinges on resistance to treatment as a primary mechanism of treatment failure. Perez-Velazquez and Rejniak (Drug-Induced Resistance in Micrometastases: Analysis of Spatio-Temporal Cell Lineages) use a hybrid agent-based computational model to investigate the role of the tumor microenvironment in the evolution of resistance in micrometastases. The authors examine the dynamics of drug distribution and oxygen gradients in the tissue to simulate response at single cell resolution. Their modeling suggests that resistant cell clones need not exist prior to treatment administration, rather, that they may emerge, and even be induced, by the treatment itself. The authors conclude that successful treatment strategies may mirror those from the field of microbial resistance to antibiotics, where the goal of treatment is to mitigate the emergence of resistance, rather than complete eradication of the cancer cells. Agent-based modeling helps gain insight into dynamics often not directly observable and is a valuable approach in cancer research.

Accurate and quick prediction of stable peptide binding to Class I HLAs is important for designing immunotherapies but also for evaluating the immunogenicity of different peptides. Abella et al. (Large-Scale Structure-Based Prediction of Stable Peptide Binding to Class I HLAs Using Random Forests) attempted to predict stable binding for class I HLAs from pHLA structures using machine learning. The structures are generated from a large set of pHLA sequences using the authors’ previously developed method named the Anchored Peptide-MHC Ensemble Generator. These structures are then transformed into feature vectors for training using random forest classifier to distinguish binders from non-binders. The model achieves competitive performance using significantly less data when compared to other popular sequence-based, pan-allele models with the advantage of interpretability.

Tao et al. (Neural Network Deconvolution Method for Resolving Pathway-Level Progression of Tumor Clonal Expression Programs With Application to Breast Cancer Brain Metastases) develop computational methods to infer clonal heterogeneity and dynamics across progression stages via deconvolution and clonal phylogeny reconstruction of pathway-level expression signatures in order to reconstruct how these processes might influence average changes in genomic signatures over progression. In this work, the authors show, via application to a study of gene expression in a collection of matched breast primary tumor and metastatic samples, that the method can infer coarse-grained substructure and stromal infiltration across the metastatic transition. Their results suggest that genomic changes observed in metastasis, such as gain of the ErbB signaling pathway, are likely caused by early events in clonal evolution followed by expansion of minor clonal populations in metastasis, a finding that may have translational implications for early detection or prevention of metastasis.

Molecular disease subtypes characterized by relevant clinical differences, such as survival, are difficult to differentiate. Tran et al. (A Novel Method for Cancer Subtyping and Risk Prediction Using Consensus Factor Analysis) have introduced a new method based on Consensus Factor Analysis (CFA) for disease subtyping and risk assessment using multi-omics data. The new method is capable of exploiting complementary signals available in different types of data to improve the subtypes. Using a large dataset of 30 different cancers from TCGA, it outperformed existing approaches in discovering novel subtypes with significantly different survival profiles. In particular, the authors demonstrated that the new method was able to predict risk scores that are highly correlated with vital status and survival probability. The accuracy of risk prediction was shown to improve as more data types were integrated.

Although most mathematical models deal with a primary cancer, metastasis is the most fatal and clinically challenging phase of cancer progression. This is particularly the case in colorectal cancer (CRC), with the added challenge that metastatic lesions often may not be detectable. To address this challenge, Hochman et al. (Metastasis Initiation Precedes Detection of Primary Cancer—Analysis of Metastasis Growth *in vivo* in a Colorectal Cancer Test Case) analyze growth rates derived from serial computed tomography (CT) scans collected from a single patient with several metastatic lesions arising from CRC. Making calculations of growth rates using three different mathematical models, the authors estimate that metastasis may have occurred 4–5 years before the primary tumor diagnosis, suggesting that metastasis may be an early event in CRC. This provocative hypothesis has potentially dramatic implications for clinical management of CRC by suggesting that the primary lesion may already have spread through the body by the time it is detected. Here, the use of mathematical modeling aided the investigators in rolling the clock back in time to calculate the likely order of events which are not otherwise clear.

One of the main contribution of the work developed in Manjunath et al. (ABC-GWAS: Functional Annotation of Estrogen Receptor-Positive Breast Cancer Genetic Variants) is the creation of the database “Analysis of Breast Cancer GWAS” (ABC-GWAS), available at http://education.knoweng.org/abc-gwas/. ABC-GWAS is an interactive database of functional annotation of estrogen receptor-positive breast cancer genome-wide association studies (GWAS) variants. This resource provides useful practical results and conceptual approaches to the functional genomics community in general and breast cancer researchers in particular. Over the past decade, hundreds of GWAS have implicated genetic variants in various diseases, including cancer. However, only a few of these variants have been functionally characterized to date, mainly because the majority of the variants reside in non-coding regions of the human genome with unknown function. A comprehensive functional annotation of the candidate variants is thus necessary to fill the gap between the correlative findings of GWAS and the development of therapeutic strategies. By integrating large-scale multi-omics datasets such as the Cancer Genome Atlas (TCGA) and the Encyclopedia of DNA Elements (ENCODE), the authors performed multivariate linear regression analysis of expression quantitative trait loci, sequence permutation test of transcription factor binding perturbation, and modeling of three-dimensional chromatin interactions to analyze the potential molecular functions of 2,813 single nucleotide variants in 93 genomic loci associated with estrogen receptor-positive breast cancer. To facilitate rapid progress in functional genomics of breast cancer, they have created ABC-GWAS. This resource includes expression quantitative trait loci, long-range chromatin interaction predictions, and transcription factor binding motif analyses to prioritize putative target genes, causal variants, and transcription factors. An embedded genome browser also facilitates convenient visualization of the GWAS loci in genomic and epigenomic context. ABC-GWAS provides an interactive visual summary of comprehensive functional characterization of estrogen receptor-positive breast cancer variants. The web resource will be useful to both computational and experimental biologists who wish to generate and test their hypotheses regarding the genetic susceptibility, etiology, and carcinogenesis of breast cancer. ABC-GWAS can also be used as a user-friendly educational resource for teaching functional genomics.

Over the last 50 years, glioblastoma has remained one of the most difficult cancers to treat. A frequent and primary cause of treatment failure in glioblastoma is drug delivery and transport across the blood-brain-barrier (BBB), which is designed to protect the brain from harmful substances. Using patient-derived xenograft models with tumor burden followed over time with bioluminescence imaging, Massey et al. (Quantifying Glioblastoma Drug Response Dynamics Incorporating Treatment Sensitivity and Blood Brain Barrier Penetrance From Experimental Data) estimate parameters for a predictive mathematical model to suggest that BBB permeability may be more important in determining response to treatment than relative sensitivity of the glioblastoma cells to treatment. This prediction underscores the challenges in treating glioblastoma and suggests that response may be improved with therapeutic approaches which do not rely on transport across the BBB. Mathematical models informed by experimental data and motivated by clinical challenges is the essence of mathematical and computational oncology.

The work developed by Dinh et al. (Application of the Moran Model in Estimating Selection Coefficient of Mutated CSF3R Clones in the Evolution of Severe Congenital Neutropenia to Myeloid Neoplasia) focuses on the transition from severe congenital neutropenia (SCN) to pre-leukemic myelodysplastic syndrome (MDS). Stochastic mathematical models have been conceived that attempt to explain the transition of SCN to MDS, in the most parsimonious way, using extensions of standard processes of population genetics and population dynamics, such as the branching and the Moran processes. The authors previously presented a hypothesis of the SCN to MDS transition, which involves directional selection and recurrent mutation, to explain the distribution of ages at onset of MDS or acute myeloid leukemia (AML). Based on experimental and clinical data and a model of human hematopoiesis, a range of probable values of the selection coefficient s and mutation rate μ have been determined. These estimates lead to predictions of the age at onset of MDS or AML, which are consistent with the clinical data. In this work, based on data extracted from published literature, we seek to provide an independent validation of these estimates. The goal of this work is twofold: 1) to determine the ballpark estimates of the selection coefficients and verify their consistency with those previously obtained and 2) to provide possible insight into the role of recurrent mutations of the G-CSF receptor in the SCN to MDS transition.

Subbalakshmi et al. (NFATc Acts as a Non-Canonical Phenotypic Stability Factor for a Hybrid Epithelial/Mesenchymal Phenotype), employ an integrated computational-experimental approach, and show that the transcription factor nuclear factor of activated T-cell (NFATc) can inhibit the process of complete epithelial–mesenchymal transition, thus stabilizing the hybrid E/M phenotype. Reversible transitions between epithelial and mesenchymal phenotypes—epithelial–mesenchymal transition (EMT) and its reverse mesenchymal–epithelial transition (MET)—form a key axis of phenotypic plasticity during metastasis and therapy resistance. Unlike previously identified phenotypic stability factors (PSFs), NFATc does not increase the mean residence time of the cells in hybrid E/M phenotypes, as shown by stochastic simulations; rather it enables the co-existence of epithelial, mesenchymal and hybrid E/M phenotypes and transitions among them. Clinical data suggests the effect of NFATc on patient survival in a tissue-specific or context-dependent manner. The results of Subbalakshmi et al. suggest that NFATc behaves as a non-canonical PSF for a hybrid E/M phenotype.

Liu et al. (Identification and Validation of Two Lung Adenocarcinoma-Development Characteristic Gene Sets for Diagnosing Lung Adenocarcinoma and Predicting Prognosis) identify and validate two Lung adenocarcinoma (LUAD)-development characteristic gene sets that can be used for diagnostics and prognosis. Lung adenocarcinoma (LUAD) is one of the main types of lung cancer. LUAD has low early diagnosis rate, poor late prognosis, and high mortality. The study identified 84 genes that were associated with LUAD survival and named as LUAD-unfavorable gene set. 39 genes were associated with LUAD survival and named as LUAD-favorable gene set. The LUAD-unfavorable genes were significantly involved in p53 signaling pathway, Oocyte meiosis, and Cell cycle. The study was conducted on 512 LUADs from The Cancer Genome Atlas and validated and data sets from Gene Expression Omnibus. Functional enrichment analysis was used to explore the potential biological functions of LUAD-unfavorable genes.

In an effort to enable novel, quantitative measures of the tumor microenvironment, Mi et al. (Digital Pathology Analysis Quantifies Spatial Heterogeneity of CD3, CD4, CD8, CD20, and FoxP3 Immune Markers in Triple-Negative Breast Cancer) developed a computational platform and workflow for digital pathology analysis. Using triple-negative breast cancer (TNBC) as an example, the authors demonstrate how their analysis platform can automatically characterize important features from a digital pathology slide, including the invasive front, central tumor, and normal tissue. This automated analysis is critical in order to quantify spatial heterogeneity of prognostic markers in TNBC, including immune cell density and local tumor immuno-architecture. The authors show how quantitative analyses generated from their workflow can be associated with treatment outcomes to predict response and also to inform mechanistic computational models which can use these spatial maps as inputs for calibration or validation. Quantitative spatial analyses such as those provided by this tool are essential to extract the most information from precious rare clinical samples, and also critically important for predictive mathematical and computational models.

In Stiehl et al. (Computational Reconstruction of Clonal Hierarchies From Bulk Sequencing Data of Acute Myeloid Leukemia Samples), the authors develop a computational algorithm that allows identifying all clonal hierarchies that are compatible with bulk variant allele frequencies measured in a patient sample. The clonal hierarchies represent descendance relations between the different clones and reveal the order in which mutations have been acquired. The proposed computational approach is tested using single cell sequencing data that allow comparing the outcome of the algorithm with the true structure of the clonal hierarchy. The authors investigate which problems occur during reconstruction of clonal hierarchies from bulk sequencing data. The algorithm proposed by the authors provides a tool to better understand the ambiguity of such reconstructions and their sensitivity to measurement errors. Their results suggest that in many cases only a small number of possible hierarchies fits the bulk data. This implies that bulk sequencing data can be used to obtain insights in clonal evolution.

